# Near-infrared spectroscopy of blood plasma with chemometrics towards HIV discrimination during pregnancy

**DOI:** 10.1038/s41598-021-02105-5

**Published:** 2021-11-19

**Authors:** Daniel L. D. Freitas, Ana F. S. Peres, Lidiane G. Silva, João V. M. Mariz, Marcos G. Santos, Rayanne S. P. Morais, Camilo L. M. Morais, Francis L. Martin, Daniel A. V. Pascoal, Juliana D. de A. S. Camargo, Janaina C. O. Crispim, Kassio M. G. Lima

**Affiliations:** 1 Biological Chemistry and Chemometrics, Institute of Chemistry, Federal University of Rio Grande do Norte, Natal, 5072-970 Brazil; 2 Maternity School Januário Cicco, Federal University of Rio Grande do Norte, Natal, 59072-970 Brazil; 3 Department of Clinical and Toxicological Analysis, Federal University of Rio Grande do Norte, Natal, 59072-970 Brazil; 4 Brain Institute, Federal University of Rio Grande do Norte, Natal, 59056-450 Brazil; 5 School of Pharmacy and Biomedical Sciences, University of Central Lancashire, Preston, PR1 2HE UK; 6 Biocel Ltd, Hull, HU10 7TS UK; 7 Instituto de Química, Universidade Federal do Rio Grande do Norte, Natal, RN 59012-310 Brazil

**Keywords:** Near-infrared spectroscopy, Computational models

## Abstract

Prevention of mother-to-child transmission programs have been one of the hallmarks of success in the fight against HIV/AIDS. In Brazil, access to antiretroviral therapy (ART) during pregnancy has increased, leading to a reduction in new infections among children. Currently, lifelong ART is available to all pregnant, however yet challenges remain in eliminating mother-to-child transmission. In this paper, we focus on the role of near-infrared (NIR) spectroscopy to analyse blood plasma samples of pregnant women with HIV infection to differentiate pregnant women without HIV infection. Seventy-seven samples (39 HIV-infected patient and 38 healthy control samples) were analysed. Multivariate classification of resultant NIR spectra facilitated diagnostic segregation of both sample categories in a fast and non-destructive fashion, generating good accuracy, sensitivity and specificity. This method is simple and low-cost, and can be easily adapted to point-of-care screening, which can be essential to monitor pregnancy risks in remote locations or in the developing world. Therefore, it opens a new perspective to investigate vertical transmission (VT). The approach described here, can be useful for the identification and exploration of VT under various pathophysiological conditions of maternal HIV. These findings demonstrate, for the first time, the potential of NIR spectroscopy combined with multivariate analysis as a screening tool for fast and low-cost HIV detection.

## Introduction

HIV has many routes of transmission including from mother-to-child, which its called vertical transmission (VT). Worldwide, the World Health Organization (WHO) has estimated that some 1.3 million pregnant women are infected with HIV^1^, while in Brazil, 125,144 cases have been diagnosed between 2000 and 2019, according to the epidemiologic bulletin of the Brazil’s Health Ministry^2^. Among the pregnant women carrying HIV, 15–45% will transmit the virus to their child if diagnosis and adequate treatment are not employed^3^. This form of infectivity has great relevance and requires a great degree of attention from health organizations due to its undesirable consequences to the mother or child, such as abortion, stillbirth, neonatal death, premature birth, low birth weight, amongst others^4^.

Early diagnosis and treatment with antiretrovirals are measures that exhibit great potential towards reducing vertical transmission to < 5%^3^. Therefore, public health organizations are constantly pushing for more diagnosis, with an increase of 21% in the number of prenatal diagnoses in the last 10 years^5^. However, studies indicate that in Brazil only around 15% of pregnant women fully comply with their prenatal appointments schedule and necessary exams.

The development of a novel tools for the diagnosis of different diseases is extremely important, mostly when they affect pregnant women, as it is the case in which VT is capable of harming both mother and the fetus. New methodologies based on spectrometric techniques are an alternative for metabolomic screening of biofluids. Mass spectrometry and nuclear magnetic resonance (NMR) are the most common spectrometric techniques due to their relatively good sensitivity and specificity to detect diseases, although they carry an elevated cost and require a complex experimental setup^6^. Near-infrared (NIR) spectroscopy is a spectrometric technique that explores high-energy vibrational modes of molecular chemical bonds, generating a sample spectrochemical spectrum in the region between 750 to 2500 nm^7–9^. This technique employs relatively simple and low-cost instrumentation when compared to mass spectrometry, NMR or even mid-infrared spectroscopy, and is widely available in portable handheld devices for in-field analysis. In addition to the simple instrumentation, this technique is non-destructive and also reagent-free, thus experimental measurements can be performed quickly and with no or minimal sample preparation.

Therefore, there is a need for accurate and low-cost techniques for VT detection. NIR together with chemometric methods has played an increasingly important role in the field of medical and biological analysis, through quickly detecting pathological conditions, even at very early stages. Previous studies have demonstrated the clinical applications of using NIR spectroscopy in biological samples such as to detect Alzheimer’s disease in blood plasma with 93% of accuracy, 88% of sensibility and 96% of specificity^9^; to differentiate strains of *Klebsiella pneumonia* producing and non-producing carbapenemase^10^; and for the identification of *Aedes aegypti* mosquitoes infected by the Zika virus^11^. It was also used with HIV, however, with others goals that were not the discrimination between infected and healthy groups^7^.

A previous study of our group showed that it was possible to differentiate groups of pregnant women living with HIV from those who were not infected by the virus using the ATR-FTIR technique (Attenuated Total Reflection Fourier-Transform Infrared spectroscopy)^12^. Herein, NIR spectroscopy is employed to distinguish HIV-infected pregnant women compared to healthy pregnant controls (without co-morbidities). Towards this, different chemometric techniques of multivariate classification were tested with the spectral dataset in order to optimize diagnostic results. In this study, our aim was to utilize NIR to differentiate groups of pregnant women with HIV from pregnant women without it.

## Results

### Statistical analysis of clinical data

Demographic and epidemiological data of the participants can be observed in Table 1.

**Table 1:** Patients characteristics. *OR* odds ratio, *TI* trust interval. Significance p < 0.05 are highlighted in bold.

Variables	Group		p value^a^	Total	OR (TI 95%)
	Living with HIV	Healthy pregnant			
N, %	39 (50.6)	38 (49.4)		77 (100.0)	
Age, years	29 ± 6	31 ± 6	0.085	30 ± 6	–
**Education, n (%)**					
Middle school	27 (69.2)	17 (44.7)	**0.030**	44 (57.1)	2.78 (1.09–7.07)
High school or superior	12 (30.8)	21 (55.3)		33 (42.9)	Ref.
**Current smoking, n (%)**					
Yes	3 (7.7)	0 (0.0)	0.240	3 (3.9)	–
No	36 (92.3)	38 (100.0)		74 (96.1)	–
**History of smoke, n (%)**					
Yes	14 (35.9)	15 (39.5)	0.746	29 (37.7)	0.86 (0.34–2.16)
No	25 (64.1)	23 (60.5)		48 (62.3)	Ref
**Drug use, n (%)**					
Yes	10 (25.6)	3 (7.9)	**0.038**	13 (16.9)	4.02 (1.01–16.00)
No	29 (74.4)	35 (92.1)		64 (83.1)	Ref.
Gestacional age, weeks	22 ± 7	24 ± 7	0.262	23 ± 7	–
**Pregnancies, n (%)**					
More ore qual to 4 pregnancies	12 (30.8)	9 (23.7)	0.485	21 (27.3)	1.43 (0.52–3.94)
Until 3 pregnancies	27 (69.2)	29 (76.3)		56 (72.7)	Ref.
**Births, n (%)**					
None	10 (25.6)	7 (18.4)	0.445	17 (22.1)	1.53 (0.51–4.55)
One or more births	29 (74.4)	31 (81.6)		60 (77.9)	Ref.
**(%)**					
One or more abortions	12 (30.8)	8 (21.1)	0.331	20 (26.0)	1.67 (0.59–4.69)
None	27 (69.2)	30 (78.9)		57 (74.0)	Ref.

Abortions, n (%) in bold.

There was a significant association between the HIV diagnosis and the level of schooling, χ^2^ (1) = 4.715, p < 0.05. The proportion of pregnant women with education level no higher than middle school was superior in the HIV group (69.2%) when compared with the control group (44.7%) (Table 1).

The patients with education level until middle school had a chance 2.78 times higher of having HIV (OR = 2.78; IC95%: 1.09–7.07) when compared to the patients who completed high school or a superior degree (Table 1).

There was a significant association between the HIV diagnosis and the use of drugs, χ^2^ (1) = 4.319, p < 0.05. The proportion of pregnant women that use drugs was superior in the HIV group (25.6%) when compared to the control group (7.9%) (Table 1).

The patients who use drugs had a chance approximately 4 times higher of having HIV (OR = 4.02; IC95%: 1.01–16.00) when compared to the patients who don’t use drugs (Table 1). No significant association was observed between the CD4+/CD8+ ratio and the maximum viral load (ρ = − 0.346, p = 0.206). Furthermore, the CD4+ cell count showed a negative correlation with the maximum viral load (ρ = − 0.642, p = 0.010).

### NIR spectroscopy

**Figure 1:**
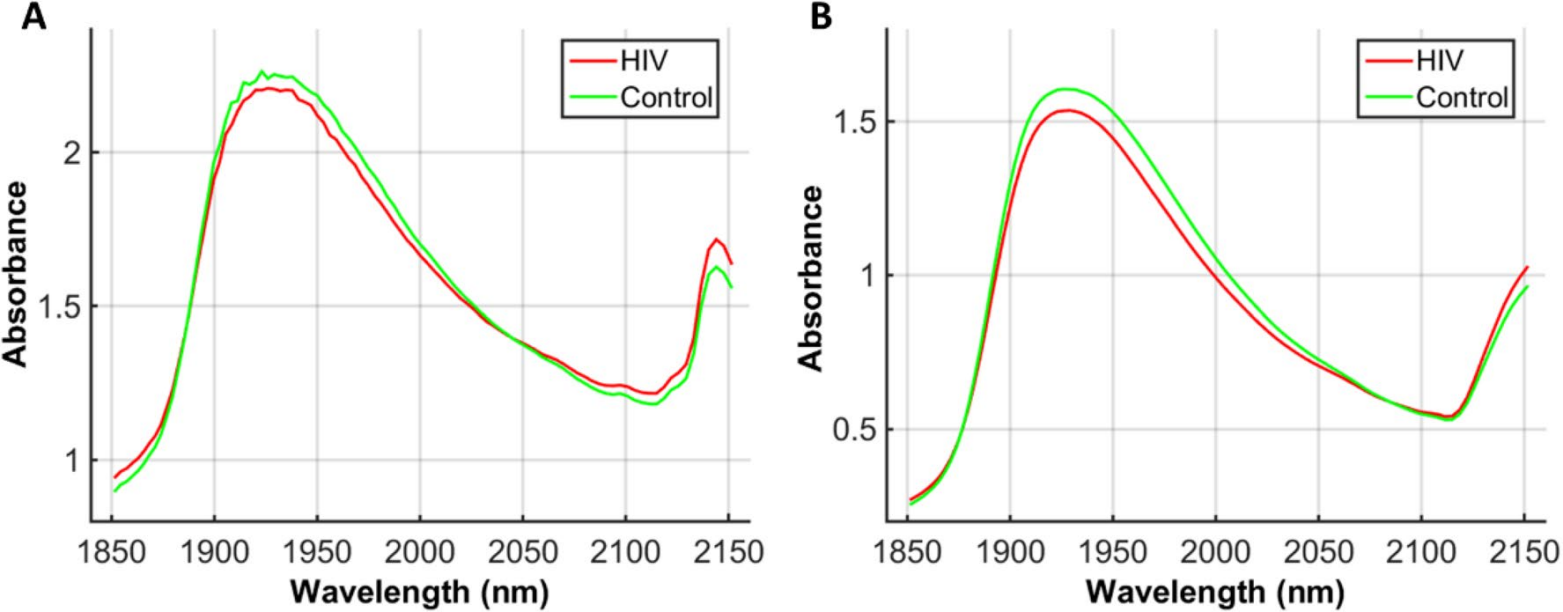
Average NIR spectra. (**A**) Raw spectra; (**B**) pre-processed (SG smoothing and baseline correction) spectra for the HIV-infected pregnant group (HIV) and healthy pregnant controls (Control).

NIR spectroscopy is a valuable tool capable of analysing different types of diseases by measuring biologically-derived samples. Herein, NIR spectroscopy was employed to detect HIV-infected blood plasma samples spectra of pregnant patients, where metrics such as diagnostic accuracy, sensitivity and specificity were calculated. Seventy-seven blood plasma samples were analysed, with 39 samples originating from HIV-infected pregnant women and 38 from healthy pregnant controls. Three spectra were collected per sample, resulting in a total of 231 spectra. The spectra were cut in the region between 1850 to 2150 nm, responsible for biomolecular-derived spectrochemical signatures. The average raw spectrum for each group of sample is depicted in Fig. 1A. To reduce noise, the raw spectral data were pre-processed by Savitzky–Golay (SG) smoothing and baseline correction (Fig. 1B). There is a high degree of superposition between spectral features among categories; consequently, multivariate analysis tools are necessary to distinguish the categories.

The spectral data were divided into training (70%) and test (30%) sets using the Kennard–Stone (KS) sample selection algorithm. To predict whether pregnant women are affected by HIV, it is necessary to use supervised classification models capable of extracting spectral features that differentiate the HIV-infected pregnant category spectra from those of healthy pregnant controls. Several supervised classification techniques were tested to discriminate the data; their performances are depicted in Table 2.

**Table 2:** Quality parameters calculated for the test set using different supervised classification algorithms to distinguish HIV-infected pregnant women and healthy pregnant controls. *AC* accuracy in %, *SENS* sensitivity in %, *SPEC* specificity in %. The best algorithm (GA-QDA) is highlighted in bold.

Model	AC (%)	SENS (%)	SPEC (%)
PCA-LDA	74	83	64
PCA-QDA	70	92	46
PCA-SVM	74	83	64
SPA-LDA	74	83	64
SPA-QDA	78	83	73
SPA-SVM	78	83	73
GA-LDA	83	83	82
**GA-QDA**	**87**	**83**	**91**
GA-SVM	74	75	73

**Figure 2:**
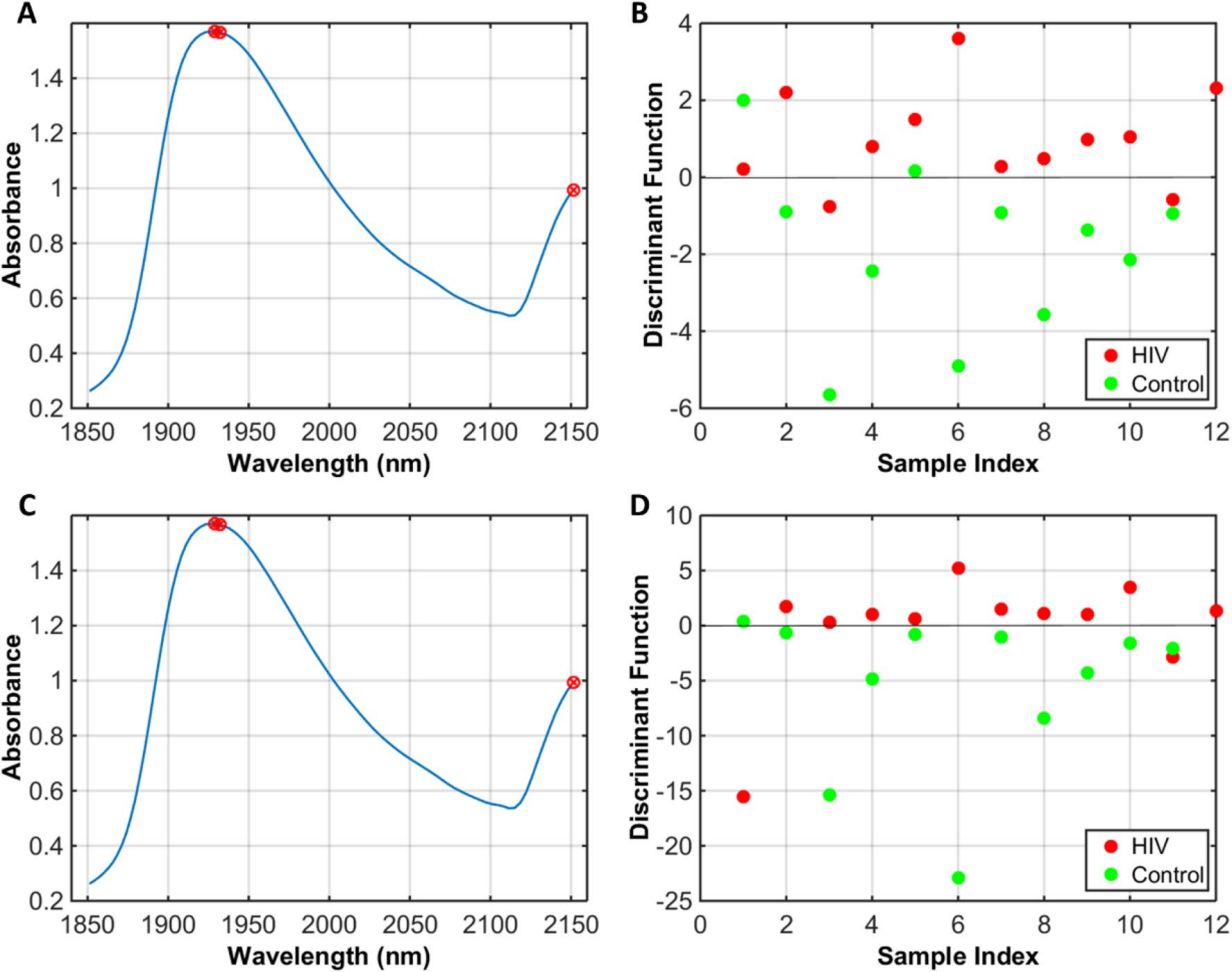
GA-LDA and GA-QDA results. (**A**) Selected variables by GA-LDA; (**B**) discriminant function in the test set for GA-LDA; (**C**) selected variables by GA-QDA; (**D**) discriminant function in the test set for GA-QDA. *HIV* HIV-infected pregnant group, *Control* healthy pregnant controls.

The best discrimination results were found for GA-QDA (accuracy of 87%), followed by GA-LDA (accuracy of 83%). The GA-LDA model selected 3 variables for category discrimination: 1929 nm, 1932 nm and 2151 nm (Fig. 2A); with a discriminant function plot showing 4 samples misclassified in the test set (Fig. 2B). GA-QDA also selected the same 3 variables (Fig. 2C), but with a more powerful discriminant function whereby 3 samples are misclassified in the test set (Fig. 2D). Three spectral wavelengths were responsible for class separation based on GA-LDA/QDA (1929, 1932 and 2151 nm).

The QDA algorithm performs better than LDA when categories have different covariance structures, since LDA assumes the categories having similar covariance matrices; hence, using a pooled covariance matrix for calculation, while QDA models each category covariance matrix separately^13^. Thus, GA-QDA is the best algorithm considering the analysis of complex sample matrices with different internal variances. The first two variables selected by GA-QDA (1929 and 1932 nm) are related to OH stretching in carbohydrates, while the variable at 2151 relates to C=O and C–N stretching in proteins^14,15^.

## Discussion

There are few studies that relate HIV and the search for new tools that will predict the possible diagnosis of HIV. To our knowledge, our group was first to use the Near-infrared spectroscopy with multivariate classification to analyze the blood plasma collected from pregnant women with HIV, where we observed towards HIV discrimination during pregnancy. In a previous study, our group found a similar result using the ATR-FTIR technique, where we were able to discriminate the groups with and without HIV^12^.

In this study, 77 blood plasma samples taken from pregnant women were analysed using NIR spectroscopy to detect HIV-infected patients. Different chemometric algorithms were tested for category discrimination, but the best classification performance was obtained with GA-QDA. HIV-infected samples were discriminated from the healthy control category with 87% accuracy, 83% sensitivity and 91% specificity using 3 spectral wavelengths selected by the genetic algorithm: 1929, 1932 and 2151 nm. The absorbances at 1929 nm and 1932 nm represent OH stretching of carbohydrates and the absorbance at 2151 nm the C=O and C–N stretching of proteins^14,16^.

According to the variables selected by the GA, we can imply that there was a slightly difference in the proteins and carbohydrates content between the tested groups. As known, blood plasma is mainly composed of water; however, there is a small proportion of proteins (albumin, fibrinogen and immunoglobulins), metabolites, carbohydrates, lipids, among other molecules^6^. This composition normally has pre-establish reference values, however, infections can generate metabolic chances that alter these values.

Among the variables selected by GA-QDA, overall the absorbance at 2151 nm (proteins) was found to be slightly higher in the HIV-infected category. Despite the metabolic alterations caused by virus infections, in this case by HIV, are not yet well understood in literature, it can be assumed that the increase of proteins in the HIV-infected samples are due to the direct action of the virus in the cell, where hyperactivation of the immunologic system caused by the HIV generates more immunoglobulins to fight the infection^17^.

The NIR spectroscopy is an analytical technique with small chemical specificity due to the high degree of overlapping of many spectral features; thus, pure biomarker identification solely based on this technique is not feasible. Although we managed to separate the groups successfully, further studies are necessary to generate a spectral pattern that is characteristic of patients with HIV. Thus, new tests with patients that are not pregnant are not in antiretroviral therapy and patients with other viral infections are very important to the validation of the technique as a possible diagnostic tool.

## Methods

### Design of study and population

This is a nested case–control study that was conducted in a Reference Obstetrics Center for AIDS at the School Maternity Januario Cicco (MEJC), located in Natal, Rio Grande do Norte, Brazil, between March 2017 and May 2019. A total of 39 pregnant women living with HIV were recruited, all a single pregnancy and gestational age between 10 and 38 weeks. The patients were selected spontaneously during their prenatal care at the Infectious Diseases Clinic at this reference maternity. All pregnant women living with HIV were monitored by an infectologist and an obstetrician. Only participants with complete clinical information were included in the analysis. Subjects were excluded if they had chronic medical conditions, including hypertension, type 2 diabetes mellitus, or heart or kidney diseases.

The study was approved by the Ethics Committee of the Federal University of Rio Grande do Norte under the protocol number 1.808.891. Written informed consent was obtained from every participant. All procedures were performed in compliance with the Declaration of Helsinki.

### Clinical measurements

Clinical data were collected by medical record review. Women living with HIV were categorized according to Antiretroviral Therapy (ART) exposure during their prenatal follow-up. Clinical, socio-epidemiological and laboratorial characteristics were summarized and submitted to a statistical analysis. We examined counts of CD4 cells, CD8 cells and CD4/CD8 ratios that are summarized in Table 3.

**Table 3:** Clinical variables of the pregnant patients infected with HIV (n = 39). The categorical data are expressed by absolute (n) and relative (%) frequencies. The continuous data are expressed by median and percentiles 25 and 75 and by mean and standard deviation.

Variables	HIV group
**Viral load, n (%)**	
Undetectable	21 (53.8)
Detectable	15 (38.5)
No information	3 (7.7)
Viral load, mm^8^	3.423 (311–8.885)
CD4+, cells (n = 36)	447 (328–804)
CD8+, cells (n = 36)	715 ± 272

### Healthy pregnant control group

This study includes 38 healthy pregnant women who attended a low-risk maternity hospital. The pregnant women were between 19 and 44 years old with a gestational age between 10 and 38 weeks. All were HIV negative and did not present with any other co-morbidity.

### Blood plasma analysis by NIR

A blood sample from each patient was collected in EDTA tubes and then submitted to centrifugation for 10 min at 1500 rpm at room temperature to obtain blood plasma for subsequent spectroscopic analysis. Maternal serum samples were collected at the time of clinically indicated blood tests and store at − 80 °C for research purpose.

### NIR spectroscopy

Plasma aliquots (stored frozen) were left to thaw for several minutes at room temperature before spectrochemical measurement. Measurements were performed in a random order without the analyst having a prior knowledge of the samples’ category. An equal number of samples were analysed per day. Before analysis, the plasma samples were homogenised for 1 min in a portable vortex mixer (Gilson Inc., USA), and then 25 μL of plasma were collected for each sample using a micropipette. The 25 μL plasma volume was then transferred to a clean enzyme-linked immunosorbent assay (ELISA) microplate (96 wells, U-type bottom). Spectrochemical measurements were carried out using an ARCoptix FT-NIR Rocket spectrometer (ARCoptix S.A., Switzerland) in the 900–2600 nm range using a fibre optic positioned onto each ELISA microwell in transflectance mode. Three replica spectra were collected per sample using an ambient air spectrum as background.

### Computational analysis

The spectral data were organized in a matrix form, where the rows contained the sample spectrum and columns contained the spectral variables (absorbance intensities for each wavelength). The data were processed using MATLAB R2014b environment (MathWorks Inc., USA) using PLS Toolbox version 7.9.3 (Eigenvector Research Inc., USA) and lab-made routines.

For supervised classification, the samples’ spectra were divided into training (70%, n = 54 [27 HIV-infected, 27 healthy controls]) and test sets (30%, n = 23 [12 HIV-infected, 11 healthy controls]) using the Kennard–Stone (KS) algorithm^18^. The training set is used for model construction and the test set for model validation. The models were built by combining feature extraction/selection algorithms with discriminant analysis. Principal component analysis (PCA)^19^ was used for feature extraction, while successive projections algorithm (SPA)^20^ and genetic algorithm (GA)^21^ were used for feature selection. Discriminant analysis was performed by linear discriminant analysis (LDA) and quadratic discriminant analysis (QDA) combined with PCA (PCA-LDA/QDA), SPA (SPA-LDA/QDA) and GA (GA-LDA/QDA). These algorithms were tested independently in order to find the best classification model.

For PCA-LDA/QDA, the PCA scores are used as input variables for LDA or QDA^13^. In SPA-LDA/QDA or GA-LDA/QDA, the selected variables by SPA or GA are used as input variables for LDA or QDA^22^. The spectral variables are selected in SPA and GA by minimising the cost function G according to the following equation:

1 $${\text{G}} = \frac{1}{{{\text{N}}_{{\text{V}}} }}\mathop \sum \limits_{{{\text{n}} = 1}}^{{{\text{N}}_{{\text{V}}} }} {\text{g}}_{{\text{n}}}$$

where ${\text{N}}_{{\text{V}}}$ represents the number of validation samples and ${\text{g}}_{{\text{n}}}$ is calculated as follows:

2 $${\text{g}}_{{\text{n}}} = \frac{{{\text{r}}^{2} ({\text{x}}_{{\text{n}}} ,\;{\text{m}}_{{{\text{I}}({\text{n}})}} )}}{{\min_{{{\text{I}}({\text{m}}) \ne {\text{I}}({\text{n}})}} {\text{r}}^{2} ({\text{X}}_{{\text{n}}} ,\;{\text{m}}_{{{\text{I}}({\text{m}})}} )}}$$

where ${\text{r}}^{2} ({\text{x}}_{{\text{n}}} ,\;{\text{m}}_{{{\text{I}}({\text{n}})}} )$ is the squared Mahalanobis distance between the object x_n_ and the centre of its true category ${\text{m}}_{{{\text{I}}({\text{n}})}}$; and ${\text{r}}^{2} ({\text{X}}_{{\text{n}}} ,\;{\text{m}}_{{{\text{I}}({\text{m}})}} )$ is the squared Mahalanobis distance between the object ${\text{x}}_{{\text{n}}}$ and the centre of the closest wrong category ${\text{m}}_{{{\text{I}}({\text{m}})}}$. GA was performed using 100 generations with 200 chromosomes each; and the mutation and cross-over probabilities were set at 10% and 60%, respectively.

The LDA (${\text{L}}_{{{\text{ik}}}}$) and QDA (${\text{Q}}_{{{\text{ik}}}}$) classification scores are calculated in a non-Bayesian form as follows^13,23^:

3 $${\text{L}}_{{{\text{ik}}}} = \left( {{\text{x}}_{{\text{i}}} - {\overline{\text{x}}}_{{\text{k}}} } \right)^{{\text{T}}} {\text{C}}_{{{\text{pooled}}}}^{ - 1} \left( {{\text{x}}_{{\text{i}}} - {\overline{\text{x}}}_{{\text{k}}} } \right)$$

4 $${\text{Q}}_{{{\text{ik}}}} = \left( {{\text{x}}_{{\text{i}}} - {\overline{\text{x}}}_{{\text{k}}} } \right)^{{\text{T}}} {\text{C}}_{{\text{k}}}^{ - 1} \left( {{\text{x}}_{{\text{i}}} - {\overline{\text{x}}}_{{\text{k}}} } \right)$$

where x_i_ is a vector containing the input variables for sample i; ${\overline{\text{x}}}_{{\text{k}}}$ is the mean spectrum of category k; ${\text{C}}_{{{\text{pooled}}}}$ is the pooled covariance matrix; and C_k_ is the variance–covariance matrix for category k.

### Model validation

The models validation performances were evaluated by calculating the accuracy (AC), sensitivity (SENS) and specificity (SPEC) for the test set. AC represents the total number of samples correctly classified; SENS represents the proportion of positives correctly classified; and SPEC represents the proportion of negatives correctly classified. These parameters are calculated as follows^24^:

5 $${\text{AC}}\; (\% ) = \left[ {\left( {{\text{TP}} + {\text{TN}}} \right){/}\left( {{\text{TP}} + {\text{FP}} + {\text{TN}} + {\text{FN}}} \right)} \right] \times 100$$

6 $${\text{SENS}}\; (\% ) = \left[ {{\text{TP/}}\left( {{\text{TP}} + {\text{FN}}} \right)} \right] \times 100$$

7 $${\text{SPEC}}\; (\% ) = \left[ {{\text{TN/}}\left( {{\text{TN}} + {\text{FP}}} \right)} \right] \times 100$$

where AC stands for accuracy, SENS for sensitivity, SPEC for specificity, TP for true positives, TN for true negatives, FP for false positives, and FN for false negatives.

### Statistical analysis

Descriptive analyses were conducted on the sociodemographic, clinical and biological data of the participants. For each categorical and continuous variable, data are reported as proportions or mean (with standard deviation) or median with interquartile range (IQR) respectively. Shapiro–Wilk normality test was applied to verify the adherence of the continuous variables to the normal distribution. A descriptive analysis of the adherent variables to the normal distribution was performed by mean and standard deviation (mean ± SD). The analysis was realized through absolute and relative frequencies to the categorical variables. The Student *t* test to independent samples was applied to the continuous variables that showed normality. The Chi-Square test was used to analyze the association between the HIV diagnosis and the categorical variables. In case of expected frequencies below five, it was applied the Fisher exact test. The odds ratio with trust interval of 95% was calculated to the binary categorical variables. The Spearman correlation was executed to evaluate the association between the CD4+/CD8+ ratio to the variables CD4+, CD8+ and the maximum viral load. The same correlation was also executed to associate the CD4+ cell count to the maximum viral load. The significance level of 5% was adopted to all the analysis.

### Ethical standards

All procedures were performed in compliance with the Declaration of Helsinki.
